# A Novel Method for Full-Section Assessment of High-Speed Railway Subgrade Compaction Quality Based on ML-Interval Prediction Theory

**DOI:** 10.3390/s24113661

**Published:** 2024-06-05

**Authors:** Zhixing Deng, Wubin Wang, Linrong Xu, Hao Bai, Hao Tang

**Affiliations:** 1 Department of Civil Engineering, Central South University, Changsha 410075, China; dzx_civil@163.com (Z.D.);; 2 National Engineering Research Center of Geological Disaster Prevention Technology in Land Transportation, Southwest Jiaotong University, Chengdu 611731, China; 3 Sichuan Expressway Construction & Development Group Co., Ltd., Chengdu 610041, China

**Keywords:** subgrade compaction quality, *ρ_dmax_* prediction, machine learning, interval prediction theory, Bootstrap

## Abstract

The high-speed railway subgrade compaction quality is controlled by the compaction degree (*K*), with the maximum dry density (*ρ_dmax_*) serving as a crucial indicator for its calculation. The current mechanisms and methods for determining the *ρ_d_*_max_ still suffer from uncertainties, inefficiencies, and lack of intelligence. These deficiencies can lead to insufficient assessments for the high-speed railway subgrade compaction quality, further impacting the operational safety of high-speed railways. In this paper, a novel method for full-section assessment of high-speed railway subgrade compaction quality based on ML-interval prediction theory is proposed. Firstly, based on indoor vibration compaction tests, a method for determining the *ρ_d_*_max_ based on the dynamic stiffness *K_rb_* turning point is proposed. Secondly, the Pso-OptimalML-Adaboost (POA) model for predicting *ρ_d_*_max_ is determined based on three typical machine learning (ML) algorithms, which are back propagation neural network (BPNN), support vector regression (SVR)*,* and random forest (RF). Thirdly, the interval prediction theory is introduced to quantify the uncertainty in *ρ_d_*_max_ prediction. Finally, based on the Bootstrap-POA-ANN interval prediction model and spatial interpolation algorithms, the interval distribution of *ρ_d_*_max_ across the full-section can be determined, and a model for full-section assessment of compaction quality is developed based on the compaction standard (95%). Moreover, the proposed method is applied to determine the optimal compaction thicknesses (*H*_0_), within the station subgrade test section in the southwest region. The results indicate that: (1) The PSO-BPNN-AdaBoost model performs better in the accuracy and error metrics, which is selected as the POA model for predicting *ρ_d_*_max_. (2) The Bootstrap-POA-ANN interval prediction model for *ρ_d_*_max_ can construct clear and reliable prediction intervals. (3) The model for full-section assessment of compaction quality can provide the full-section distribution interval for *K*. Comparing the *H*_0_ of 50~60 cm and 60~70 cm, the compaction quality is better with the *H*_0_ of 40~50 cm. The research findings can provide effective techniques for assessing the compaction quality of high-speed railway subgrades.

## 1. Introduction

The high-speed railway subgrade compaction quality is controlled by the compaction degree (*K*), with the maximum dry density (*ρ_d_*_max_) serving as a crucial indicator for its calculation [1,2]. The current mechanisms and methods for determining the *ρ_d_*_max_ contribute to the determination of *ρ*_dmax_ for coarse-grained soil filler in subgrades, but they still suffer from uncertainties, inefficiencies, and lack of intelligence [3,4]. These deficiencies can lead to insufficient assessments for the high-speed railway subgrade compaction quality, which will reduce the stability and strength of the subgrade structure and may trigger uneven settlement, track unevenness, and other diseases in the later operation process [5], seriously impacting the operation safety of high-speed railways.

*K* is obtained by dividing the measured dry density (*ρ_d_*) by the *ρ_d_*_max_ determined by the indoor compaction method [6]. Currently, there are many indoor compaction methods for determining the *ρ_d_*_max_, including Marshall compaction, rotary compaction, heavy hammer compaction, and vibratory compaction [7,8], which are used to carry out indoor compaction tests of asphalt mixtures, crushed concrete, fine-grained soils, or coarse-grained soil fills, respectively, to simulate the compaction effect of a field roller on the compacted soil. As a typical compaction method, vibration compaction has become the most efficient method to determine the *ρ_d_*_max_ of coarse-grained soil fillers for high-speed railway subgrades [9]. Nevertheless, there is still a lack of foundation on the optimal compaction time to accurately determine the *ρ_d_*_max_ of coarse-grained soil fillers for high-speed railway subgrades. Ye et al. [3] set the vibration time at 360 s and developed a mathematical model to fit the relationship between vibration compaction time and *ρ_d_*. Wang et al. [4] explored the effect of vibration time on *ρ_d_* by vibration compaction tests and indicated the optimal vibration time was 60 s. Furthermore, according to the water conservancy specification [10], the optimal vibration time was 480 s. In summary, the *ρ_d_* increases with vibration time, and the rate of increase gradually decreases. The optimal vibration time is summarized to be within 60~480 s, but the *ρ_d_*_max_ can not be determined accurately, which leads to errors in the assessment of compaction quality. Consequently, it is necessary to propose a novel method for determining the *ρ_d_*_max_ in vibratory compaction.

Based on the *ρ_d_*_max_ prediction model, the *K* can be rapidly calculated. It is an important method to assess the high-speed railway subgrade compaction quality, which can save most field test workloads [11]. However, there are typical nonlinear characteristics in the prediction task of *ρ_d_*_max_. Many scholars have used simple regression models to establish the relationship between *ρ_d_*_max_ and filler parameters [12,13], but the accuracy of prediction results is still debatable. Recently, machine learning (ML) methods have been widely used due to their nonlinear mapping, high efficiency, and intelligence [14]. The main ML algorithms include three types: (1) neural network algorithms, such as artificial neural network (ANN) [15], back propagation Neural Network (BPNN) [16], recurrent neural network (RNN) [17], and long short-term memory (LSTM) [18]. (2) ML regression algorithms, such as support vector regression (SVR) [19], and ridge regression (Ridge) [20]. (3) ML tree algorithms, such as random forest (RF) [21], and decision tree (DT) [22]. Along with the continuous development and popularization of ML algorithms, the above three types of ML algorithms have been widely used in temporal prediction tasks such as electric power loads [23], financial stock prices [24], geotechnical deformations [25], as well as in non-temporal prediction tasks such as mechanical properties of materials [26], test parameters [27], and so on. Furthermore, ML provides an effective tool for the nonlinear prediction of various parameters in vibratory compaction [28], such as optimal water content prediction [29], shear strength prediction [30], and stiffness prediction [31], etc., and it also provides an efficient and intelligent method for predicting *ρ_d_*_max_. Since high-speed railways need to strictly control the requirements of compaction quality, the prediction of *ρ*_dmax_ is more demanding, but the existing ML algorithms still have three deficiencies. Firstly, the hyperparameters of ML algorithms are difficult to adjust, limiting the improvement of their prediction accuracy. Secondly, the prediction results of individual ML algorithms are stochastic, resulting in low prediction robustness and generalization performance. Finally, all existing ML algorithms can only provide *ρ_d_*_max_ point prediction results, and can not take into account the errors caused by the uncertainty issues [32,33] in the prediction process [34,35]. There must be a large deviation between the compaction quality assessment conclusions based on the *ρ_d_*_max_ prediction results with errors and the real situation. It is necessary to propose a novel method for making the assessment results of compaction quality more reliable.

Interval prediction, also known as probabilistic prediction, can be used to quantify uncertainty in point predictions by constructing prediction intervals [36]. With the increasing concern about the uncertainty of prediction models, Delta, Bayesian, MVE, Bootstrap, and LUBE methods for constructing prediction intervals have been proposed [37,38]. They also have been successfully applied to many fields such as finance [39], wind power [40], energy [41], etc. The Bootstrap method is a nonparametric statistical method based on resampling, which has the advantage of relying only on the original observation data [42]. Because of the unique advantages of the Bootstrap method [43], it has been applied in engineering fields, such as dam deformation prediction [44] and slope deformation prediction [45]. In addition, it can also provide a new method for modeling the statistical distribution under the condition of limited data in vibration compaction. Hence, it is necessary to introduce the Bootstrap method to modify the uncertainty issues based on the existing high-precision *ML* algorithms and further realize the accurate high-speed railway subgrade compaction quality assessment.

This study aims to address the issues of uncertainties, inefficiencies, and lack of intelligence in the mechanisms and methods for determining the *ρ_d_*_max_. To this end, a novel method for full-section assessment of high-speed railway subgrade compaction quality based on ML-interval prediction theory is proposed. The structure of this paper is organized as follows: Section 2 provides a detailed introduction to the novel method. Section 3 shows the results of the *ρ_d_*_max_ point prediction and interval prediction. A case study is provided in Section 4, applying the *ρ_d_*_max_ interval prediction model to the compaction test section of a station in Southwest China, and further combining it with the spatial interpolation algorithm to realize the full-section assessment of high-speed railway subgrade compaction quality. Meanwhile, the optimal paving thickness *H*_0_ for subgrade compaction is determined based on assessment results. Finally, the conclusions of the study are presented in the last section.

## 2. Methodology

**Figure 1:**
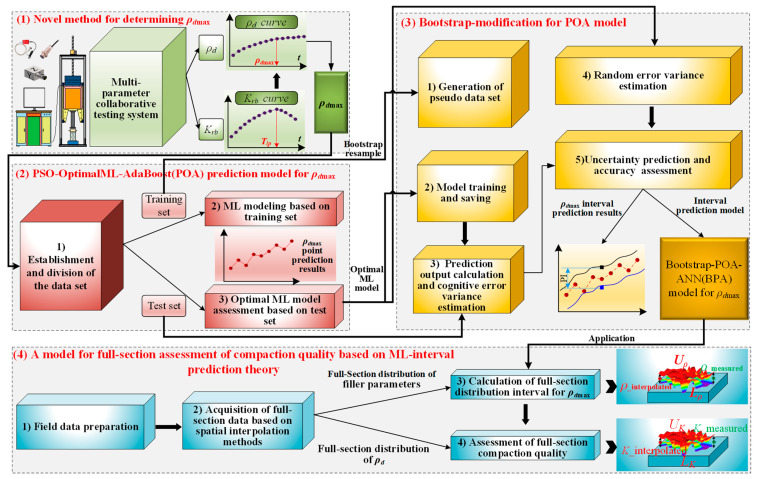
A framework for full-section assessment of high-speed railway subgrade compaction quality based on ML-interval prediction theory.

As shown in Figure 1, to address the issues of uncertainties, inefficiencies, and lack of intelligence in the mechanisms and methods for determining the *ρ_d_*_max_, the method for full-section assessment of high-speed railway subgrade compaction quality based on ML-interval prediction theory is proposed. Firstly, based on indoor vibration compaction tests and a multi-parameter collaborative testing method, a method for determining the *ρ_d_*_max_ based on the dynamic stiffness *K_rb_* turning point is proposed. Secondly, based on three typical machine learning (ML) algorithms, which are back propagation neural network (BPNN), support vector regression (SVR), and random forest (RF), a PSO-ML-AdaBoost hybrid model is developed, achieving intelligent and rapid prediction of the *ρ_d_*_max_. Moreover, the PSO-OptimalML-AdaBoost (POA) model is chosen based on prediction accuracy and error, which can guarantee the prediction accuracy of *ρ_d_*_max_. Thirdly, the interval prediction theory(Bootstrap) is introduced to quantify the uncertainty in *ρ_d_*_max_ prediction. The Bootstrap-based POA model is applied to estimate the *ρ_d_*_max_ predicted output and variance of cognitive error. Subsequently, the ANN model is applied to estimate the variance of random error. Finally, based on the Bootstrap-POA-ANN interval prediction model and spatial interpolation algorithms, the interval distribution of *ρ_d_*_max_ across the full-section is determined, and a model for full-section assessment of compaction quality is developed based on the compaction standard (95%). It is worth noting that the final proposed model, which can be applied to the assessment of compaction quality in the construction site of high-speed highway subgrade, and the case study is described in Section 4.

### 2.1. Novel Method for Determining ρ_dmax_

**Figure 2:**
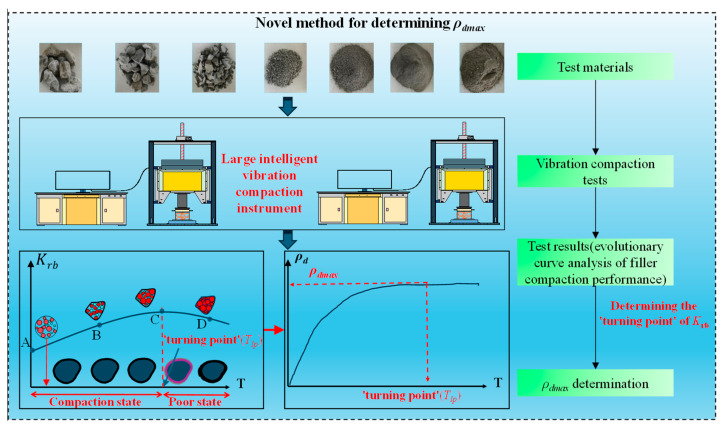
Novel method for determining *ρ*_dmax.._ The progression from A to B to C represents the gradual·densification of the particles in the compacted·state. The progression from C to D represents the gradual deterioration of the particles after optimal compaction time.

There is still no agreement on determining the optimal time for vibratory compaction. Additionally, an increase in the *ρ_d_*_max_ of the coarse-grained soil fillers does not indicate an increase in its mechanical strength, and there is a lack of an indicator to assess the quality of subgrade filler compaction at the level of mechanical properties [46]. It is worth noting that the concept of the “turning point” is proposed in the impact and rotary compaction methods, the moment of stabilization of the filler structure in the vibratory compaction process, and stopping the compaction at the moment corresponding to the “turning point” can guarantee the quality of the compaction [47,48]. Meanwhile, this concept can be introduced into vibratory compaction, and based on the “turning point” characteristic, it can be used to represent the critical moment of deterioration of the compaction factor *K* to further determine the optimal compaction time and to control the over-compaction. Hence, for the indoor vibration compaction test of high-speed railway subgrade coarse-grained soil fillers, a novel method of determining the *ρ_d_*_max_ by combining the dry density *ρ_d_* and the dynamic stiffness *K_rb_* is proposed [49]. The specific process is shown in Figure 2, which mainly includes four steps: test materials, vibration compaction tests, test results, and *ρ*_dmax_ determination. The test filler is from the site of the Guangzhou-Zhanjiang High-Speed Railway, and subsequent content will elaborate on the process of steps 2–3.

#### 2.1.1. Multi-Parameter Collaborative Testing System for Compaction Quality

**Figure 3:**
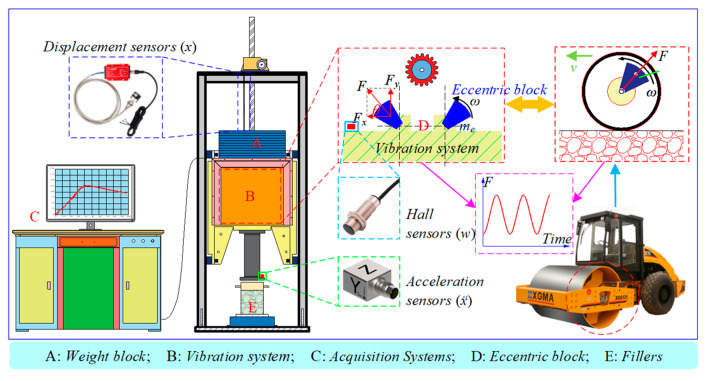
Large intelligent vibration compaction instrument.

As shown in Figure 3, vibration compaction tests were conducted using the improved large intelligent vibration compaction instrument. Compared to conventional vibration compaction instruments, it marks the first integration of displacement sensors, hall sensors, acceleration sensors, and others. Equations (1) and (2) are used to calculate the real-time output of the dry density *ρ_d_* curve and dynamic stiffness *K_rb_* curve.

(1) $$\rho_{d}=\frac{mg}{\pi D_{c}^{2}\left(h_{0}-S_{n}\right)}$$

(2) $$K_{rb}=\frac{m_{e}r_{e}\omega^{2}\sin\left(\Delta \varphi \right)+m_{p}g-{m_{d}\ddot{x}|}_{\dot{x}=0}}{{x|}_{\dot{x}=0}}$$

where *m* is the mass of the fillers, *D*_c_ is the internal diameter of the compaction cylinder, *h*_0_ is the pavement thickness, *S*_n_ is the displacement rate of fillers, *m_e_* is the mass of eccentric block, *r_e_* is the eccentricity, *ω* is the rotation speed of the eccentric block, Δ*φ* is the lag phase angle obtained from the hall sensor, *m_d_* is the mass of the vibration system, *x* is the displacement of the vibration system obtained from the displacement sensor, and *ẍ* is the acceleration of the vibration system obtained from the acceleration sensor. A specific derivation of *K_rb_* is available in reference [46,49].

#### 2.1.2. Method for Determining ρ_dmax_ Based on the ‘Turning Point’ of K_rb_

The indoor vibratory compaction tests are carried out using the test parameters in Table 1. The optimal moisture content of each grading aggregate is determined by the test calibration method, and the inherent frequency of each level is determined by the hammer modal analysis method. To ensure the stability of the vibratory compactor, it is necessary to ensure that the ratio of the excitation force to the static mass is 1.8.

**Figure 4:**
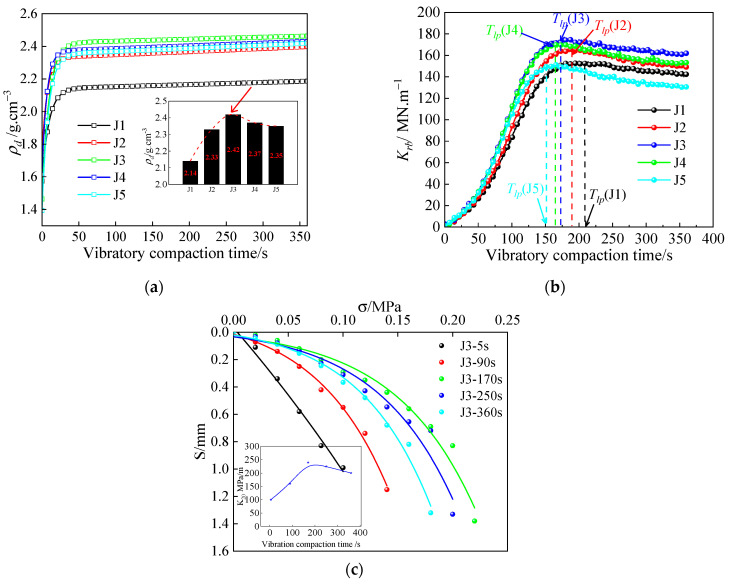
Evolution curve of *ρ_d_*, *K_rb_*, and *K*_20_ for grading aggregates: (**a**) *ρ_d_;* (**b**) *K_rb_;* and (**c**) *K*_20._

The compaction performance evolution of the five high-speed railway grading aggregates (J1–J5) is shown in Figure 4. As shown in Figure 4a, it is found that during the vibratory compaction process, the *ρ_d_* of J1–J5 all increase rapidly in the initial stage, and then grow slowly. Hence, it is difficult to determine the optimal vibratory compaction time by the change of *ρ_d_*. As shown in Figure 4b, there is a ‘turning point’ of *K_rb_* during vibratory compaction, and the *K_rb_* of J1–J5 all show a rapid increase and then a slow decrease in evolution. Hence, the vibratory compaction time corresponding to the ‘turning point’ of *K_rb_* can be used as the optimal compaction time (*T_lp_*) [2], and further determining the *ρ_d_*_max_ of the high-speed railway grading aggregates on the *ρ_d_* compaction curve. 

Taking the J3 as an example, the *K_rb_* reaches a peak value of 173.68 MN/m at optimal compaction time (*T_lp_* = 170.2 s), with a *ρ_d_* of 2.42 g/cm^3^ considered as *ρ_dmax_* at this time. To further validate the proposed method, an investigation is conducted to identify if there exists an inflection point for another mechanical index, the modified foundation coefficient (*K*_20_) [49], during the vibratory compaction process. It is also examined whether this inflection point aligns with *T_lp_*. Using J3 as a reference, indoor plate load tests are performed at various compaction stages (5, 90, 170, 250, and 360 s), revealing the change in *K*_20_ over time as shown in Figure 4c. At *T_lp_* = 170.2 s, *K*_20_ reaches its maximum value of 240.03 Mpa/m and gradually decreases beyond *T*_lp_. This indicates that the method for determining *ρ*_dmax_ based on the “turning point” of *K_rb_* is valid.

In summary, solely relying on the physical index *ρ_d_* for assessing compaction quality may lead to the inability to determine the optimal vibration compaction time. The mechanical index *K_rb_* serves as a valuable supplement to improve the assessment system for vibration compaction. Additionally, *T_lp_* can be used as a control standard for assessing compaction quality. Furthermore, *ρ_dmax_* can be determined on the compacted density curve.

### 2.2. POA Prediction Model for ρ_dmax_

**Figure 5:**
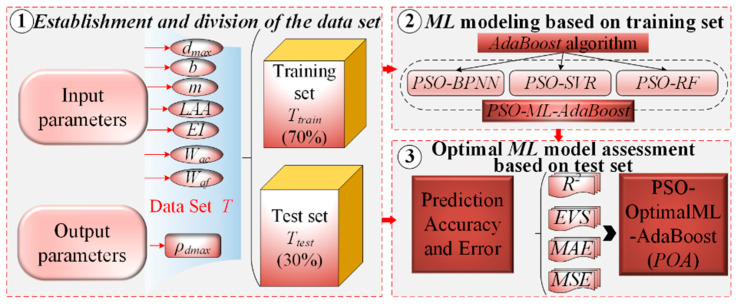
*POA* prediction model for *ρ_dmax._*

Figure 5 shows the establishment process of the POA prediction model for *ρ_d_*_max_, which primarily includes three steps: establishment and division of the data set, ML modeling based on the training set, and optimal ML model assessment based on the test set.

Step 1: establishment and division of the data set

Referring to previous studies [2,50], the GRA feature selection algorithm based on GRA defines seven features, maximum particle size (*d*_max_), grading parameter (*b*), grading parameter (*m*), Los Angeles abrasion (*LAA*), coarse particle slenderness ratio (*EI*), coarse aggregate water absorption (*W*_ac_), and fine aggregate water absorption (*W*_af_) as the main controlling features affecting *p_dmax_*. Hence, these seven features are used as input features for the POA model, and *ρ_d_*_max_ is used as the output parameter, which in turn constructs the dataset. The data set is divided into training and test sets at a 7:3 ratio [51]. The ML model is developed using the training set, and the POA model is determined using the test set.

Step 2: ML modeling based on training set

**Figure 6:**
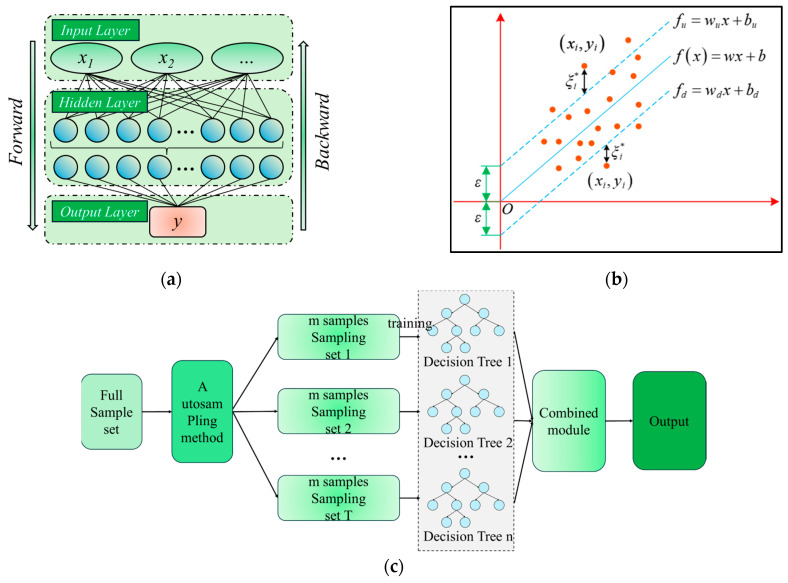
Three selected ML algorithms: (**a**) BPNN; (**b**) SVR; and (**c**) RF.

Figure 6 shows the network structure of the three selected ML algorithms. BPNN is an optimization algorithm based on gradient descent. The core idea is to calculate the error between the output layer of the network and the true value and make it possible to minimize the total error of the network. SVR excels in solving non-linear problems and can adapt to various data distributions. It creates a “margin band” on both sides of the linear function to determine loss calculation based on the data-margin band relationship. RF is an ML tree model that constructs and combines the outputs of multiple Decision Tree to improve the prediction performance of regression problems. Considering the characteristics and advantages of these ML algorithms, in vibratory compaction parameters prediction, the most frequently used and representative ML algorithms in recent years are BPNN, SVR, and RF [52,53,54]. Hence, three typical ML algorithms—BPNN, SVR, and RF—are selected for *ρ_d_*_max_ prediction.

Controlling the hyperparameters of ML algorithms is challenging, which limits the improvement of prediction accuracy. Traditional methods of hyperparameter tuning are limited to manual tuning, which is not only inefficient, but also prone to local optima. To address this, the PSO algorithm [55] is introduced for adaptive hyperparameter adjustment. However, the PSO-ML model still faces challenges such as overfitting and parameter randomization, leading to lower predictive robustness and generalization performance. To address this, the AdaBoost ensemble algorithm [56] is introduced and a PSO-ML-AdaBoost hybrid prediction model for *ρ_d_*_max_ is developed, which includes PSO-BPNN-AdaBoost, PSO-SVR-AdaBoost, and PSO-RF-AdaBoost.

Step 3: optimal ML model assessment based on test set

To determine the *POA* model, the performance of each *ML* model is assessed in terms of prediction accuracy and error metrics, including *R*^2^, *EVS*, *MAE*, and *MSE*.

### 2.3. Bootstrap-Modification for POA Model

#### 2.3.1. Sources of Uncertainty in ρ_dmax_ Predictions

**Figure 7:**
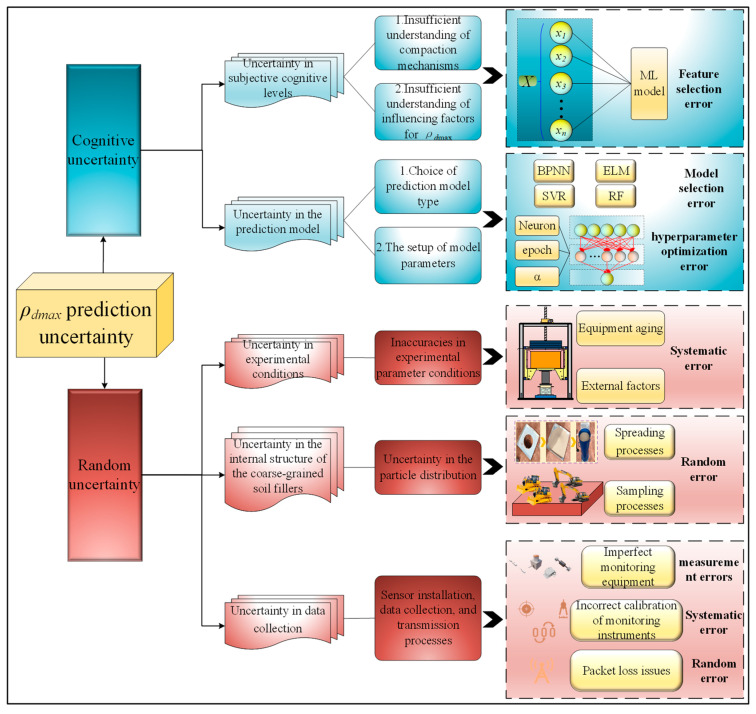
Sources of prediction uncertainty.

While the POA model guarantees the accuracy of *ρ_d_*_max_ prediction, there exist limits in the point prediction results provided by it. The POA model cannot account for errors caused by uncertainties in the prediction process, which significantly impact the reliability and credibility of the *ρ_d_*_max_ prediction results. Uncertainties in *ρ_d_*_max_ prediction include both cognitive and random uncertainty, as shown in Figure 7.

(1) Cognitive uncertainty

Cognitive uncertainty is influenced by subjective cognitive levels and the prediction model, including two primary forms: ① Due to an insufficient understanding of compaction mechanisms or influencing factors for *ρ_d_*_max_, some crucial input parameters in the prediction may be overlooked. This significantly impacts the structure of the POA prediction model, substantially increasing the uncertainty of the prediction results. ② Uncertainty of the prediction results can also be caused by the choice of prediction model type and the setup of model parameters. For example, in the model hyperparameter optimization process, challenges may emerge in selecting the optimization algorithm and determining the optimization criteria. These factors unavoidably introduce uncertainty in the subsequent prediction results.

(2) Random uncertainty

Random uncertainty, also known as noise uncertainty, is affected by experimental noise data. It includes uncertainties related to experimental conditions, internal structure of the coarse-grained soil fillers, and data collection: ① Uncertainties in the vibration compaction test process are affected by inaccuracies in experimental parameter conditions, which mainly include equipment aging and external factors. ② High-speed railway subgrade coarse-grained soil fillers are defined as complex particulate materials, introducing uncertainty in the particle distribution during the sampling and spreading processes. ③ Errors are inevitably introduced during sensor installation, data collection, and transmission processes due to various conditions. For example, measurement errors may be caused by imperfect monitoring equipment, and systematic errors may be caused by incorrect calibration of monitoring instruments, and so on. Hence, the *ρ_d_*_max_ prediction model trained and tested based on this data will inevitably be influenced by data errors, resulting in uncertainty.

After identifying the sources of prediction uncertainty, we can start with cognitive uncertainty and random uncertainty, respectively, to quantify the prediction error.

#### 2.3.2. Quantification of ρ_dmax_ Prediction Uncertainty

**Figure 8:**
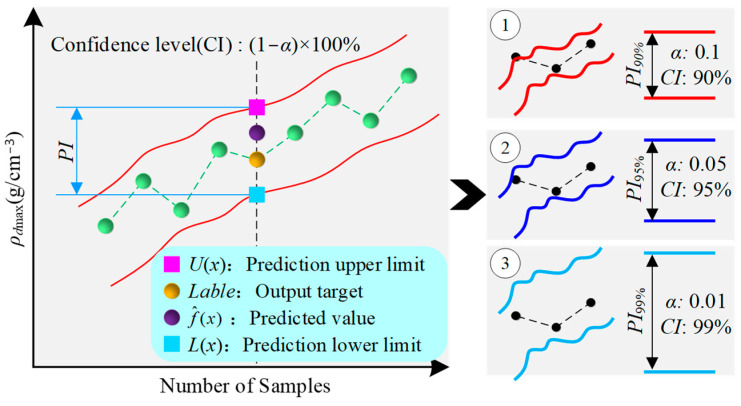
Prediction interval structure.

To further quantify the uncertainty in the *ρ_d_*_max_ prediction process, a Prediction Interval (PI) based on interval prediction theory is proposed [57]. As shown in Figure 8, a PI includes a prediction upper limit and lower limit, representing the estimated range of the predicted *ρ_d_*_max_ under a certain confidence level (*CI*) [58]. In interval prediction, the significance levels *α* and *CI* are two key parameters that control the width of the PI. The *α* are commonly set to 0.1, 0.05, and 0.01, corresponding to *CIs* of 90%, 95%, and 99% [59]. It is noted that the smaller the *α* and the larger the *CI*, the wider the width of the PI.

**Figure 9:**
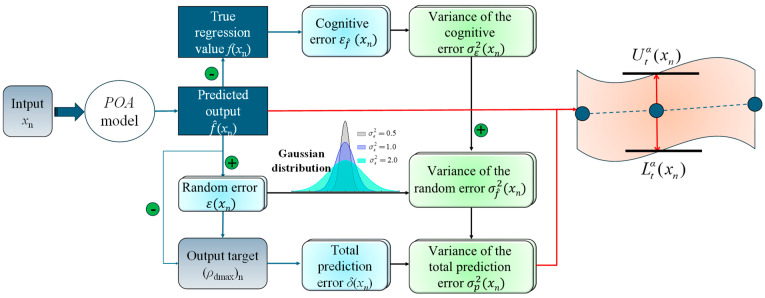
Quantification of *ρ*_dmax_ prediction uncertainty.

Assuming the data set for *ρ_d_*_max_ prediction is *T* = {(*x_n_*, (*ρ_d_*_max_)_n_)${\}}_{n=1}^{N}$, where *x_n_* represents the input parameter vector of the prediction model and (*ρ_d_*_max_)*_n_* is the corresponding output target. The process of quantifying the uncertainty in *ρ_d_*_max_ prediction to construct the PI of *ρ_dmax_* is shown in Figure 9, which consists of four main steps: determining the expression for the output target, representation of *ρ_dmax_* prediction error, calculation of the variance of total prediction error for *ρ_dmax_*, and construction of the prediction interval for *ρ_dmax_*.

(1) Determining the expression for the output target

Assuming a non-linear mapping relationship *f(x)* between the output target *ρ_dmax_* and the input *x_n_*, the output target can be represented as follows:

(3) $${\left(\rho_{d\max}\right)}_{n}=f(x_{n})+\varepsilon (x_{n})$$

where *f*(*x_n_*) is the true regression value, reflecting the main effect of input *x_n_* on output target (*ρ_d_*_max_)*_n_*. *ε*(*x_n_*) is the target observation noise (also known as random error), primarily caused by uncertainties in the *ρ_d_*_max_ experimental process. *ε*(*x_n_*) follows a Gaussian distribution with a mean of 0 and a variance of $\sigma_{\varepsilon }^{2}$(*x_n_*) [44].

(2) Representation of *ρ_dmax_* prediction error

Utilizing the POA model for *ρ_d_*_max_ regression prediction, the model is trained on the training set and predicts on the test set, generating a predicted output $\hat{f}(x_{n})$. The prediction error can be represented as:

(4) $${\left(\rho_{d\max}\right)}_{n}-\hat{f}(x_{n})=(f(x_{n})-\hat{f}(x_{n}))+\varepsilon (x_{n})$$

where ${\left(\rho_{d\max}\right)}_{n}-\hat{f}(x_{n})$ is the total prediction error *δ*(*x_n_*), and $f(x_{n})-\hat{f}(x_{n})$ is the difference between the predicted output and the true regression value, denoted as cognitive error $\varepsilon_{\hat{f}}(x_{n})$. This error mainly arises from the uncertainty in the prediction model itself.

(3) Calculation of the variance of total prediction error for *ρ_dmax_*

Before constructing the PI, it is necessary to calculate the variance of the total prediction error $\sigma_{\rho }^{2}(x_{n})$. The total prediction error includes cognitive error and random error, which are independent of each other. The variance of the total error can be represented as:

(5) $$\sigma_{\rho }^{2}(x_{n})=\sigma_{\hat{f}}^{2}(x_{n})+\sigma_{\varepsilon }^{2}(x_{n})$$

where $\sigma_{\hat{f}}^{2}(x_{n})$ is the variance of the cognitive error, and $\sigma_{\varepsilon }^{2}(x_{n})$ is the variance of the random error.

(4) Construction of the prediction interval for *ρ_dmax_*

It is obvious that the PI of *ρ_d_*_max_ is a random interval $I_{t}^{\alpha }(x_{n})$ at a given significance level of *α*:

(6) $$I_{t}^{\alpha }(x_{n})=\left[L_{t}^{\alpha }(x_{n}),U_{t}^{\alpha }(x_{n})\right]$$

where $L_{t}^{\alpha }(x_{n})$ is the prediction lower limit, $U_{t}^{\alpha }(x_{n})$ is the prediction upper limit. $L_{t}^{\alpha }(x_{n})$ and $U_{t}^{\alpha }(x_{n})$ can be calculated as:

(7) $$L_{t}^{\alpha }(x_{n})=\hat{f}(x_{n})-Z_{\alpha /2}\sqrt{\sigma_{\rho }^{2}(x_{n})}$$

(8) $$U_{t}^{\alpha }(x_{n})=\hat{f}(x_{n})+Z_{\alpha /2}\sqrt{\sigma_{\rho }^{2}(x_{n})}$$

where *Z*_α/2_ is the *α*/2 percentile of the standard normal distribution, and its value depends on *CI* [45]. *Z*_α/2_ is 1.65, 1.96, and 2.58 when the *CI* is taken as 90%, 95%, and 99%, respectively.

#### 2.3.3. Interval Prediction for ρ_dmax_ Based on POA Model

The POA model can not quantify prediction errors arising from various uncertainties in the prediction process. The main parameters for constructing the PI can be acquired by the Bootstrap method, such as prediction output $\hat{f}(x_{n})$, variance of cognitive error $\sigma_{\hat{f}}^{2}(x_{n})$, variance of random error $\sigma_{\varepsilon }^{2}(x_{n})$, and variance of total error $\sigma_{\rho }^{2}(x_{n})$ [44,45]. Hence, the Bootstrap method is used to correct the prediction error of the POA model. Then, a method for *ρ_d_*_max_ interval prediction based on the Bootstrap-POA-ANN model is proposed, which not only accurately predicts *ρ_d_*_max_, but also quantifies the uncertainty of the predicted values in the form of an interval. 

**Figure 10:**
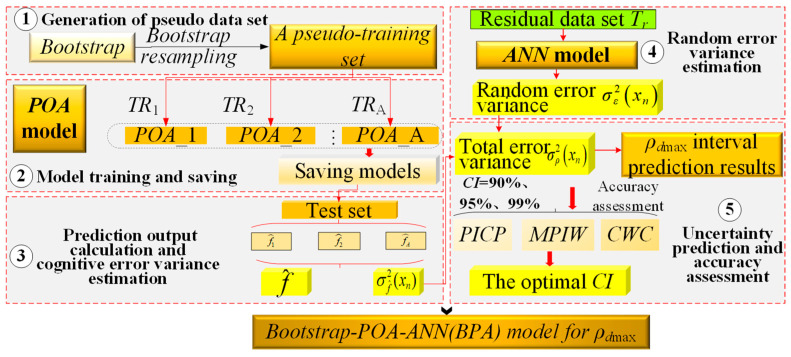
Bootstrap-modification for *ρ_dmax_* POA model.

As shown in Figure 10, the framework of *ρ_d_*_max_ interval prediction mainly includes five steps: generation of pseudo data set, model training and saving, prediction output calculation and cognitive error variance estimation, random error variance estimation, and uncertainty prediction and accuracy assessment.

(1) Generation of pseudo data set

**Figure 11:**
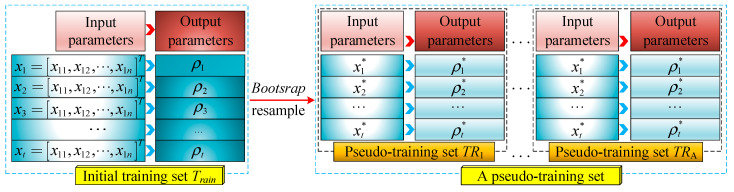
Illustration of pseudo-training set generation.

Based on the Bootstrap method, the pseudo-training set TR^*^ can be acquired by resampling *N*_train_ times (*N*_train_ is the total number of samples in the training set). Repeat these steps *A* times to complete the generation of pseudo-training set, as shown in Figure 11.

(2) Model training and saving

Maintaining the hyperparameters and structure of the POA model unchanged, the pseudo-training set is input into the POA model. Then, the POA model is executed for training and saved at the end of each training session, and these steps are repeated *A* times to obtain well-trained POA models.

(3) Prediction output calculation and cognitive error variance estimation

The test set is input into each of the *A* well-trained POA prediction models, which will yield *A* predicted *ρ_d_*_max_ results as follows:

(9) $${\hat{f}}_{a}=\left[{\hat{f}}_{a}(x_{1}),{\hat{f}}_{a}(x_{2}),\ldots {\hat{f}}_{a}(x_{n})\right],(a=1,2\ldots A)$$

Combining the results of the *A* models, an estimated value of the true regression of *ρ_d_*_max_ under small bias conditions is obtained. The calculation method is as follows:

(10) $$\hat{f}(x_{n})=\frac{1}{A}\sum_{a=1}^{A}{\hat{f}}_{a}(x_{n})$$

where $\hat{f}(x_{n})$ is the mean of the *ρ_d_*_max_ predictions from the *A* prediction models for the nth samples and is also considered as the representative value of predicted output.

The *ρ_d_*_max_ predictions from the POA models are assumed to be unbiased [60], and the variance of cognitive error can be estimated as follows:

(11) $$\sigma_{\hat{f}}^{2}(x_{n})=\frac{1}{A-1}\sum_{a=1}^{A}\left({\hat{f}}_{a}(x_{n}\right)-\hat{f}(x_{n}){)}^{2}$$

(4) Random error variance estimation

It is also necessary to estimate the variance of random error after determining the variance of cognitive error. Based on Equation (5), the variance of random error can be estimated as follows [44]:

(12) $$\sigma_{\varepsilon }^{2}(x_{n})\approx E\{{\left({\left(\rho_{d\max}\right)}_{n}-\hat{f}(x_{n})\right)}^{2}\}-\sigma_{\hat{f}}^{2}(x_{n})$$

To realize the prediction of $\sigma_{\varepsilon }^{2}(x_{n})$, based on Equation (12), a new set of squared residual sequences is reconstructed:

(13) $$r^{2}(x_{n})=\max({\left({\left(\rho_{d\max}\right)}_{n}-\hat{f}(x_{n})\right)}^{2}-\sigma_{\hat{f}}^{2}(x_{n}),0)$$

where $\hat{f}(x_{n})$ and $\sigma_{\hat{f}}^{2}(x_{n})$ can be obtained from Equations (10) and (11), respectively. *r*^2^(*x_n_*) is the squared residual sequence for predicting the *ρ_d_*_max_. By combining *r*^2^(*x_n_*) with the predicted input parameters vector *x_n_*, the squared residual data set *T*_r_^2^ can be established:

(14) $$T_{r^{2}}={\left\{(x_{n},r^{2}(x_{n}))\right\}}_{n=1}^{N}$$

Similarly, the squared residual dataset is divided in the ratio of 7:3 to obtain the squared residual training set *T*_r_^2^__train_ and the squared residual test set *T*_r_^2^__test_. Furthermore, the *T*_r_^2^__train_ can be used as a basis for developing a prediction model of the $\sigma_{\varepsilon }^{2}(x_{n})$. Referring to the established studies [61,62], the ANN model can be introduced to support the prediction of $\sigma_{\varepsilon }^{2}(x_{n})$. During the training process, the goal is to maximize the probability of observing samples from $\sigma_{\varepsilon }^{2}(x_{n})$ in *T*_r_^2^__train_. Traditional metrics such as *MSE* or *MAE* fail to achieve this goal. Hence, the concept of maximum likelihood estimation is introduced to enhance the loss function *Loss_ε_* of the ANN model [46]:

(15) $$Loss_{\varepsilon }=\frac{1}{2}\sum_{n=1}^{N_{train}}\left(\ln\sigma_{\varepsilon }^{2}(x_{n})+\frac{r^{2}(x_{n})}{\sigma_{\varepsilon }^{2}(x_{n})}\right)$$

where *ln*() is the logarithmic operation with base *e*. It is noted that to ensure the predicted output of the ANN model is always positive, the activation function of the output layer should be set to an exponential function (e.g., the exponential function, softplus function, etc.). In this study, the softplus activation function is employed. After training, the squared residual test set *T*_r_^2^__test_ is input into the model to obtain results.

(5) Uncertainty prediction and accuracy assessment

To assess the accuracy of the uncertainty prediction and to determine an optimal *CI*, metrics such as prediction interval coverage probability (*PICP*) [63], mean prediction interval width (*MPIW*) [64], and coverage width-based criterion (*CWC*) [65] are employed.

(16) $$PICP=\frac{1}{N_{\mathrm{test}}}\sum_{t=1}^{N_{\mathrm{test}}}c_{t}$$

(17) $$MPIW=\frac{1}{N_{\mathrm{test}}}\sum_{t=1}^{N_{\mathrm{test}}}\left[U_{t}^{\alpha }(x_{t}))-L_{t}^{\alpha }(x_{t}))\right]$$

(18) $$CWC=MPIW[1+\gamma (PICP)\times e^{\eta (CI-PICP)}]$$

where *N*_test_ is the total number of samples in the test set, *C_t_* and *γ*(*PICP*) are Boolean values, and *η* is the penalty parameter, which together with *CI* determines the degree of penalty.

### 2.4. A Model for Full-Section Assessment of Compaction Quality Based on ML-Interval Prediction Theory

**Figure 12:**
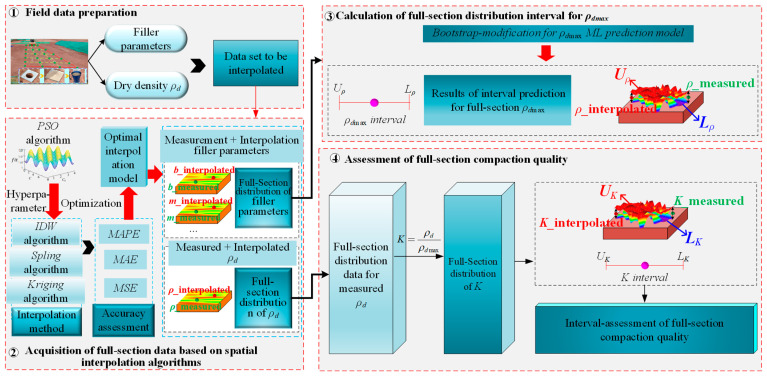
A model for the full-section assessment of compaction quality based on ML-interval prediction theory.

A *ρ_dmax_* interval prediction model based on indoor experiments is developed in Section 2.3, and this model is saved and can be applied to the engineering field. Figure 12 shows the establishment process for the full-section assessment model of compaction quality based on ML-interval prediction theory, which primarily includes four steps: field data preparation, acquisition of full-section data based on spatial interpolation algorithms, calculation of full-section distribution interval for *ρ_d_*_max_, and assessment of full-section compaction quality.

#### 2.4.1. Field Data Preparation

*K* can be obtained by the field measured *ρ_d_* and the experiment tested *ρ_d_*_max_, as shown in Equation (19). During field experiments, the *ρ_d_* of different test pit locations can be measured using the sand cone method, and *ρ_d_*_max_ can be determined through the POA model. The determination process of *ρ_dmax_* is as follows: ① The coarse-grained soil fillers from the test pit are taken back to the field laboratory, and tests on parameters are conducted such as gradation characteristics, shape, water absorption rate, crushing wear, etc. ② The filler parameters are input into the POA model to predict *ρ_d_*_max_.

(19) $$K=\frac{\rho_{d}}{\rho_{d\max}}$$

#### 2.4.2. Acquisition of Full-Section Data Based on the Spatial Interpolation Algorithm

Considering the limitation of the field experiment, it is not possible to carry out the full cross-section test, so it is necessary to combine the interpolation algorithm to enrich the established sparse measurement points. The spatial interpolation algorithm is a method of calculating spatial distribution data based on location information. The fundamental principle underlying this algorithm is the ‘First Law of Geography’, which assumes that points in closer spatial locations are more likely to share similar feature values [66]. Hence, it can be applied to the continuous processing of sparse test pit data (filler parameters, measured *ρ_d_*) to further analyze the full-section distribution patterns of field test data.

**Figure 13:**
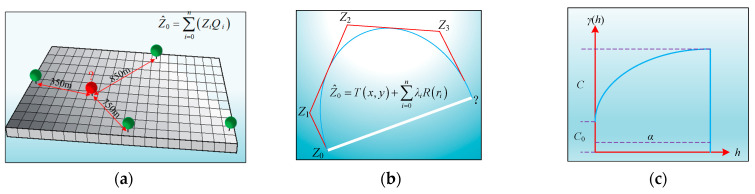
Three typical interpolation algorithms: (**a**) IDW; (**b**) Spline; (**c**) Kriging.

As shown in Figure 13, we employ three typical interpolation algorithms for calculating test pit data across the full-section: Inverse Distance Weighting (IDW) [67], Spline function interpolation (Spline) [68], and Kriging interpolation [69]. In the process of developing the interpolation model, cross-validation [70] is used to assess the interpolation accuracy and determine the optimal interpolation algorithm. Moreover, the optimal interpolation algorithm provides data support for subsequent calculation of the full-section distribution interval for *ρ_d_*_max_ and assessments of full-section compaction quality.

#### 2.4.3. Calculation of Full-Section Distribution Interval for ρ_dmax_

Once the full-section distribution of filler parameters is acquired, it is inputted into the *ρ_d_*_max_ interval prediction model based on the *Bootstrap* method, which can calculate the full-section distribution interval for *ρ_d_*_max_.

#### 2.4.4. Assessment of Full-Section Compaction Quality

Based on the obtained full-section distribution interval for *ρ_d_*_max_, the full-section distribution of *K* is calculated by combining the measured and interpolated full-section distribution data of *ρ_d_*, and achieving the assessment of full-section compaction quality.

## 3. ρ*_d_*_max_ Interval Prediction Results

### 3.1. Prediction Database for ρ_dmax_

**Figure 14:**
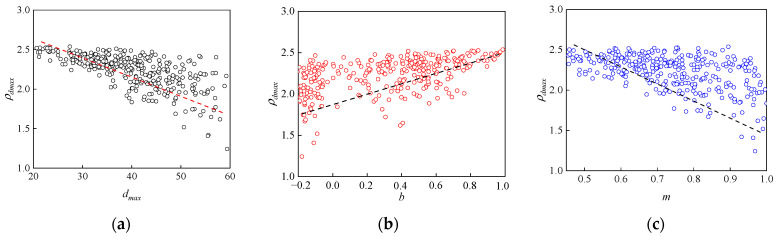

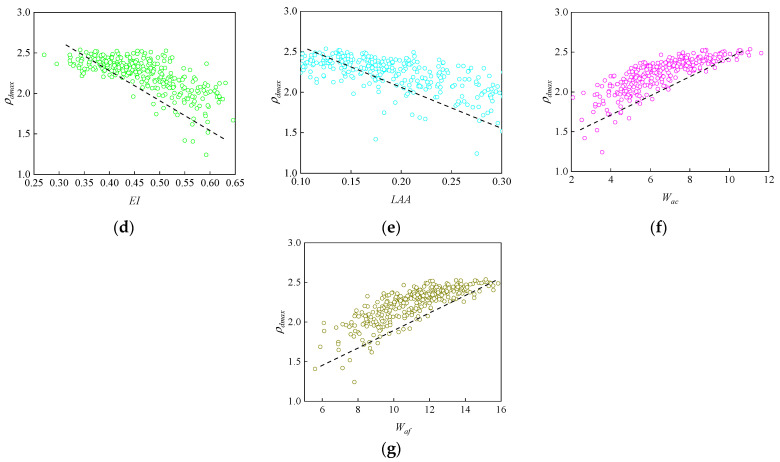
*ρ_dmax_* prediction database: (**a**) *d*_max_; (**b**) *b*; (**c**) *m*; (**d**) *EI*; (**e**) *LAA*; (**f**) *Wac*; (**g**) *Waf*.

The surface layer of the subgrade is most strongly influenced by the high-speed train loads and external environmental factors [71]. Typically, in Chinese high-speed railway subgrades, the surface layer is compacted using high-speed railway grading aggregates with good strength and deformation characteristics [72]. Inadequate compaction of the high-speed railway grading aggregates can significantly reduce the stability and strength of the subgrade structure. Therefore, this paper focuses on high-speed railway grading aggregates as the research subject for subsequent tests and analyses. As shown in Figure 14, based on the optimal vibration compaction parameters determined in the previous tests [46,49], a large number of vibration compaction tests were conducted to establish a database of the properties of high-speed railway grading aggregates and the *ρ_d_*_max_. It can be observed that the *ρ_d_*_max_ of high-speed railway grading aggregates is negatively correlated with *d_max_*, *m*, *EI*, and *LAA*, while its is positively correlated with *b*, *W_ac_*, and *W_af_*. The properties of high-speed railway grading aggregates, including *d_max_*, *m*, *b*, *EI*, *LAA*, *W_ac_*, and *W_af_*, are used as input parameters for the prediction model, with *ρ_d_*_max_ as the output parameter.

### 3.2. Determination of POA Prediction Model for ρ_dmax_

**Figure 15:**
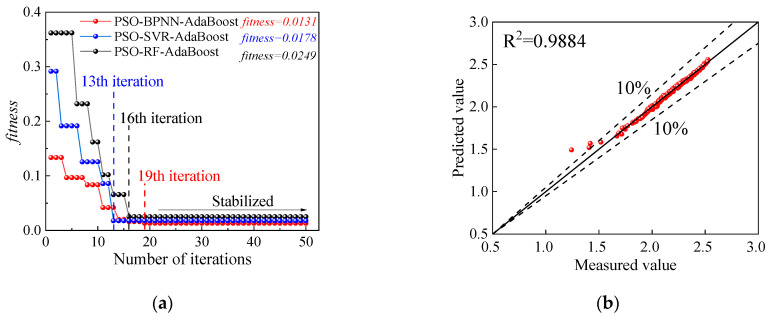

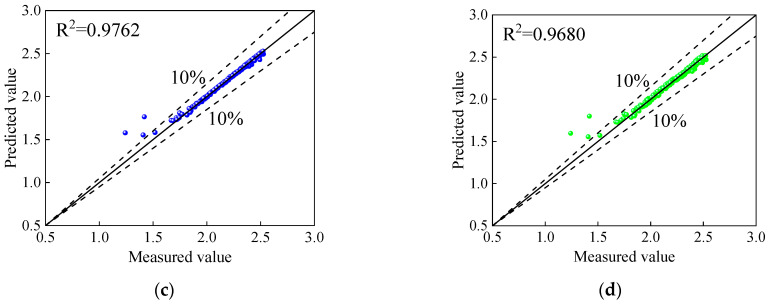
PSO parameter optimization results and fitting results on the training set: (**a**) PSO parameter optimization; (**b**) PSO-BPNN-AdaBoost; (**c**) PSO-SVR-AdaBoost; and (**d**) PSO-RF-AdaBoost.

After establishing the prediction dataset of *ρ*_dmax_, it is inputted into the PSO-ML-AdaBoost hybrid prediction model, and the PSO algorithm optimization results as well as the prediction results on the training set can be obtained at first. Figure 15a shows the PSO parameter optimization results for each model. The fitness value of each model is significantly reduced and stabilized before 20 iterations, indicating that the PSO algorithm has an advantage in improving the accuracy of the ML model. Among them, the PSO-BPNN-AdaBoost model has the lowest fitness value of 0.0131, which indicates the advantage of this model to some extent. The fitting results of each model on the training set are shown in Figure 15b–d. The models all fit well to the measured values of *ρ_d_*_max_ on the training set, with most of the points clustered within the 10% error line, indicating that the fitting errors of the models are all low. Since the prediction results on the training set only indicate the prediction ability in the process of ML model building, which can not yet reflect the generalization performance, the optimal ML model is further determined through the test set.

**Figure 16:**
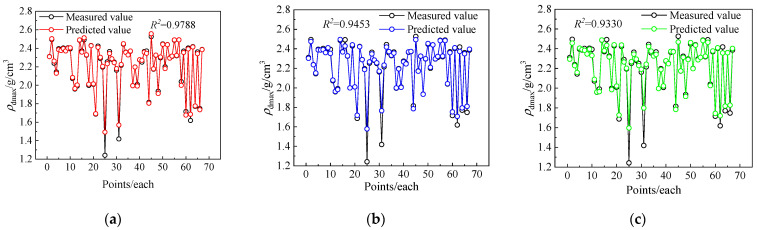
Point prediction results: (**a**) PSO-BPNN-AdaBoost; (**b**) PSO-SVR-AdaBoost; and (**c**) PSO-RF-AdaBoost.

The global distribution map of the predictions for each model on the test set is shown in Figure 16. It is obvious that all three *ML* models exhibit good performance for *ρ_d_*_max_, and the obtained predicted values align well with the overall trend of the measured values. Notably, the PSO-BPNN-AdaBoost model shows the tightest clustering around the measured curve with an *R*^2^ of 0.9788, which is mainly due to the fact that BPNN models are neural network models with strong nonlinear processing capabilities, making them capable of capturing and modeling complex nonlinear relationships. In addition, neural network models have high-dimensional data processing capabilities that enable BPNN to make accurate predictions with a large number of input variables. Second on the list is the PSO-SVR-AdaBoost model (0.9453) and lastly the PSO-RF-AdaBoost model (0.9330). The RF model has significantly lower prediction accuracy for *ρ*_dmax_ compared to the BPNN and SVR models, mainly due to the fact that it is more difficult for the *ML* tree model than the *ML* regression model to capture linear relationships and extend them beyond the training set, and more difficult for the neural network model to capture the interactions between input features. Moreover, Table 2 indicates a comparison of accuracy assessment results. The PSO-BPNN-AdaBoost model outperforms others in both prediction accuracy and error metrics. Consequently, the PSO-BPNN-AdaBoost model is chosen as the POA model for *ρ_dmax_*.

**Figure 17:**
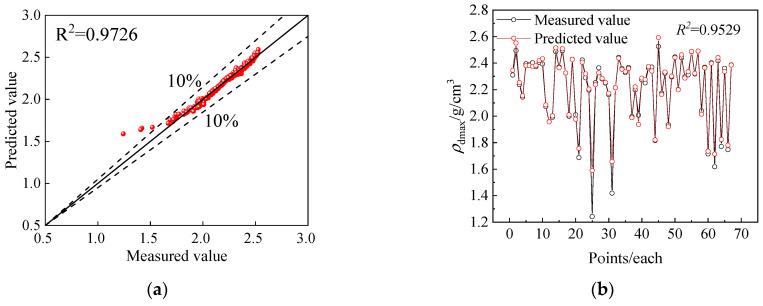
The prediction performance of PSO-BPNN model: (**a**) fitting results on the training set for PSO-BPNN; (**b**) prediction results on the training set for PSO-BPNN.

To highlight the impact of AdaBoost algorithm on the prediction results of ML models, the prediction performance of PSO-BPNN-AdaBoost model and PSO-BPNN model is compared with the BPNN model as an example, which is shown in Figure 17. Comparison with the prediction results of the PSO-BPNN-Adaboost model in Figure 15 and Figure 16 indicates that the PSO-BPNN model is less effective than the PSO-BPNN-AdaBoost model on both the training set and the test set, which to some extent proves the superiority of the AdaBoost algorithm and its influence on the prediction performance of ML models.

### 3.3. Interval Prediction Results for ρ_dmax_ Based on Bootstrap-POA-ANN Model

**Figure 18:**
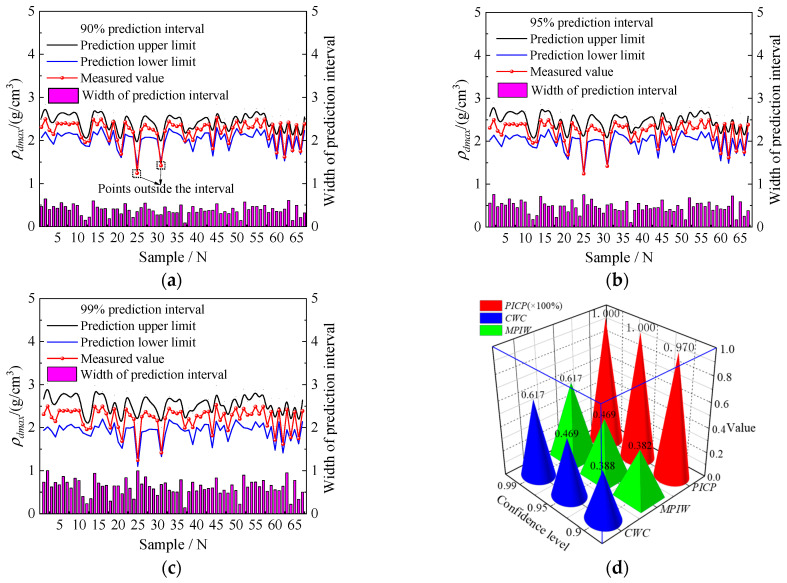
Interval prediction results: (**a**) 90% confidence level; (**b**) 95% confidence level; (**c**) 99% confidence level; and (**d**) accuracy assessment.

The results of *ρ_d_*_max_ interval prediction and accuracy assessment based on the Bootstrap-POA-ANN model are shown in Figure 18. It is clear that the prediction interval effectively encompasses the measured curve of *ρ_d_*_max_, and the measured *ρ_d_*_max_ values are largely within the obtained prediction interval, indicating the high reliability of the interval prediction results. Additionally, the overall width of the prediction interval is uniform and increases with the confidence level. As shown in Figure 18d, as the confidence level rises, both *PICP* and *CWC* increase. Moreover, under three different confidence levels, *PICP* values not only exceed the corresponding confidence levels, but also have a mean exceeding 95%, indicating the high reliability of the interval prediction results obtained with the Bootstrap-POA-ANN model.

It is important to note that a higher *PICP*, smaller *MPIW*, and *CWC* indicate higher accuracy and more reliable results [73,74]. Under a 95% confidence level, the *PICP*, *MPIW*, and *CWC* are 100%, 0.4690 g/cm^3^, and 0.4690 g/cm^3^, respectively. Although the *PICP* is higher than the 90% confidence level and equal to the 99% confidence level, both *MPIW* and *CWC* are lower than the 99% confidence level. Hence, the 95% confidence level can be selected as a core parameter in the Bootstrap-POA-ANN model for subsequent compaction quality assessment.

**Figure 19:**
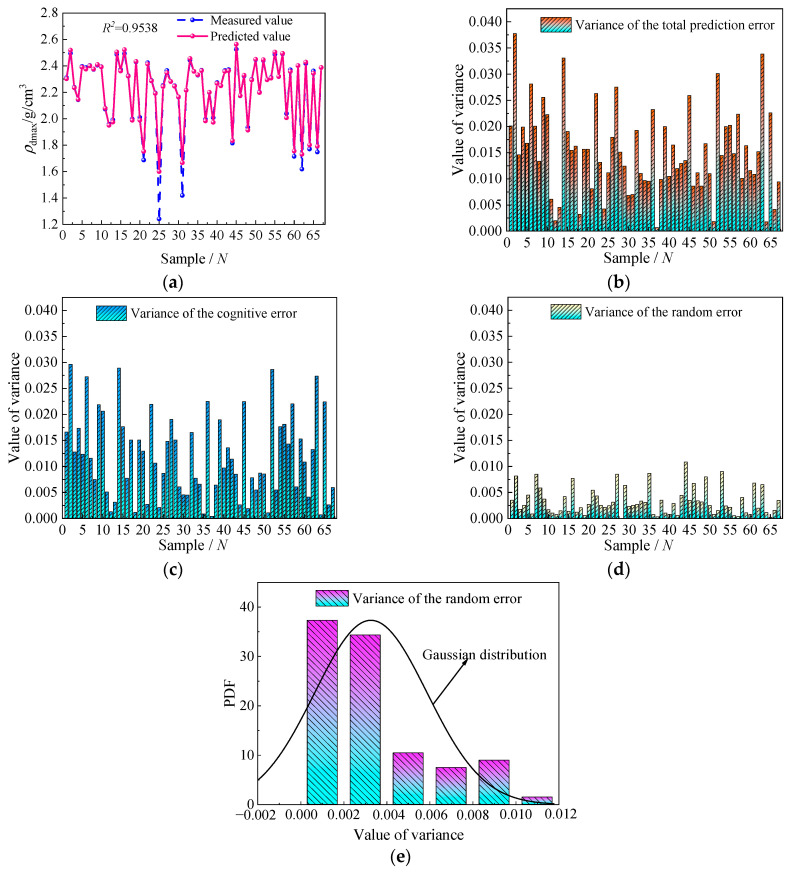
Predicted output and variance at 95% confidence level: (**a**) predicted output; (**b**) variance of the total error; (**c**) variance of the cognitive error; (**d**) variance of the random error; and (**e**) distribution of random error variance.

The prediction output and variance results obtained from the Bootstrap-POA-ANN model at the 95% confidence level are shown in Figure 19. As shown in Figure 19a, the point prediction results obtained from the Bootstrap-POA-ANN model and the *POA* model are relatively close to each other, and both can well reflect the fluctuations of the *ρ_d_*_max_ values. It is noted that the *R*^2^ of the Bootstrap-POA-ANN model is 0.9538, which is lower than the *POA* model. It mainly takes into account the unavoidable lack of input information caused by the Bootstrap method. The variance of the total prediction error $\sigma_{\rho }^{2}(x_{n})$ is shown in Figure 19b, and its value stays below 0.05. The pattern of change in the $\sigma_{\rho }^{2}(x_{n})$ is consistent with the width of the prediction intervals in Figure 18b, which is consistent with Equations (7) and (8). The variance of the cognitive error $\sigma_{\hat{f}}^{2}(x_{n})$ and the variance of the random error $\sigma_{\varepsilon }^{2}(x_{n})$ are shown in Figure 19c,d. The comparison shows that the value of the $\sigma_{\hat{f}}^{2}(x_{n})$ is much higher than the $\sigma_{\varepsilon }^{2}(x_{n})$, indicating that cognitive uncertainty accounts for a major proportion of the prediction uncertainty. The distribution of the $\sigma_{\varepsilon }^{2}(x_{n})$ satisfies the Gaussian distribution, proving the correctness of the interval prediction results, as shown in Figure 19e.

## 4. Case Study-Determining Optimal Paving Thickness H_0_ for Subgrade Compaction

### 4.1. Overview

**Figure 20:**
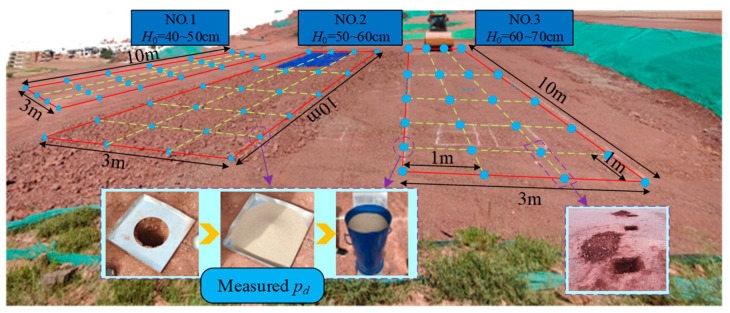
Test section.

Before large-scale paving and compaction, it is necessary to conduct a test section to optimize construction process parameters. The optimal paving thickness *H*_0_ is a crucial construction process parameter, impacting both the improvement of construction efficiency and the precise control of compaction quality. As shown in Figure 20, the model for full-section assessment of compaction quality is applied to a compaction test section in the southwest region, and the optimal paving thickness *H*_0_ is determined for this test section. Three typical test sites (length 10 m, width 3 m) are selected in the test section, with different designed compaction thicknesses (*H*_0_): low thickness (40~50 cm), medium thickness (50~60 cm), and high thickness (60~70 cm).

The vibration compaction process parameters during the experiment were as follows: the self-weight was 23 t, the working width was 2.15 m, the vibration frequency was 32 Hz, and the vibration amplitude was 1.03 mm. After compacting 4~5 times in different test sections, the surface settlement of the test section stabilized. At intervals of 1 m in each test site, test pits were selected to measure the filler parameters and *ρ_d_*.

### 4.2. Full-Section Data Based on Spatial Interpolation Algorithms

#### 4.2.1. Results of Measured ρ_d_

**Figure 21:**
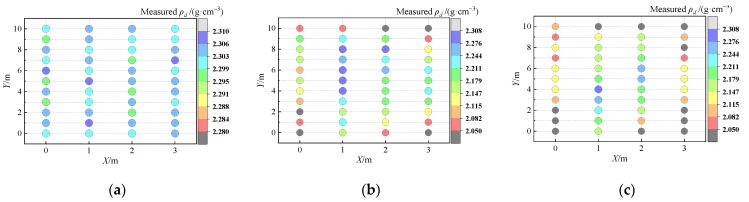
Results of measured *ρ_d_*: (**a**) 40~50 cm thickness; (**b**) 50~60 cm thickness; and (**c**) 60~70 cm thickness.

The measured *ρ_d_* results for three different *H*_0_ are shown in Figure 21. A significant increase in *ρ_d_* can be achieved by reducing the paving thickness. The distribution of measured *ρ_d_* is more uniform at lower thicknesses. However, with the increase in paving thickness, there is a decrease in *ρ_d_*. The distribution of measured *ρ_d_* becomes uneven, and the *ρ_d_* at the edge of the test section is significantly lower than that in the interior. Hence, it is necessary to strictly control the ‘weak’ areas at the edges of the compaction in field compaction quality inspection.

#### 4.2.2. Interpolation Results of ρ_d_ at 40~50 cm Thickness

**Figure 22:**
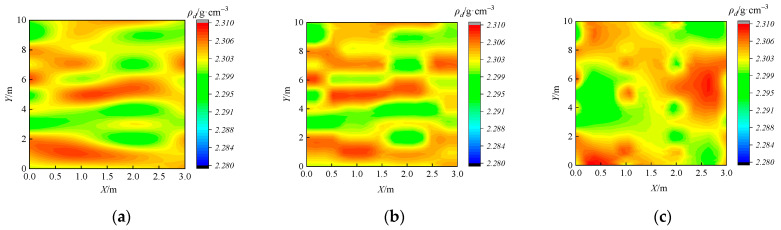
Interpolation results of *ρ*_d_ at 40 cm thickness using different interpolation algorithms: (**a**) Spline algorithm; (**b**) IDW algorithm; and (**c**) Kriging algorithm.

As shown in Figure 22, the spatial distribution of measured *ρ_d_* across the full section is obtained using different interpolation algorithms for a paving thickness of 40~50 cm. The accuracy assessment results for the three methods are shown in Table 3. The Kriging algorithm yields the minimum values for both error assessment metrics, showing the highest interpolation accuracy. Hence, Kriging is used as the optimal interpolation algorithm for measured *ρ_d_*.

#### 4.2.3. Full-Section Distribution Results of Filler Parameters at 40~50 cm Thickness

In a similar method, a screen test, water absorption test, particle shape fast scanning test, and Los Angeles abrasion test were conducted on the fillers from the test pits. Then, the parameters *d_max_*, *b*, *m*, *W_ac_*, *W_af_*, *EI*, and *LAA* can be acquired. Subsequently, the Kriging interpolation algorithm is used to obtain the full-section distribution of each filler parameter.

**Figure 23:**
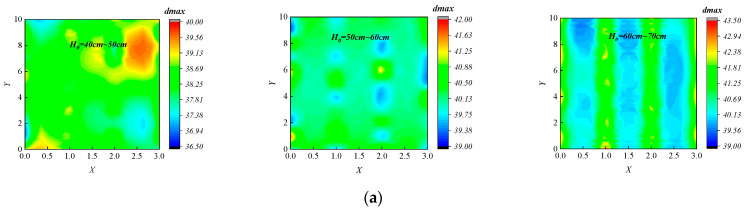

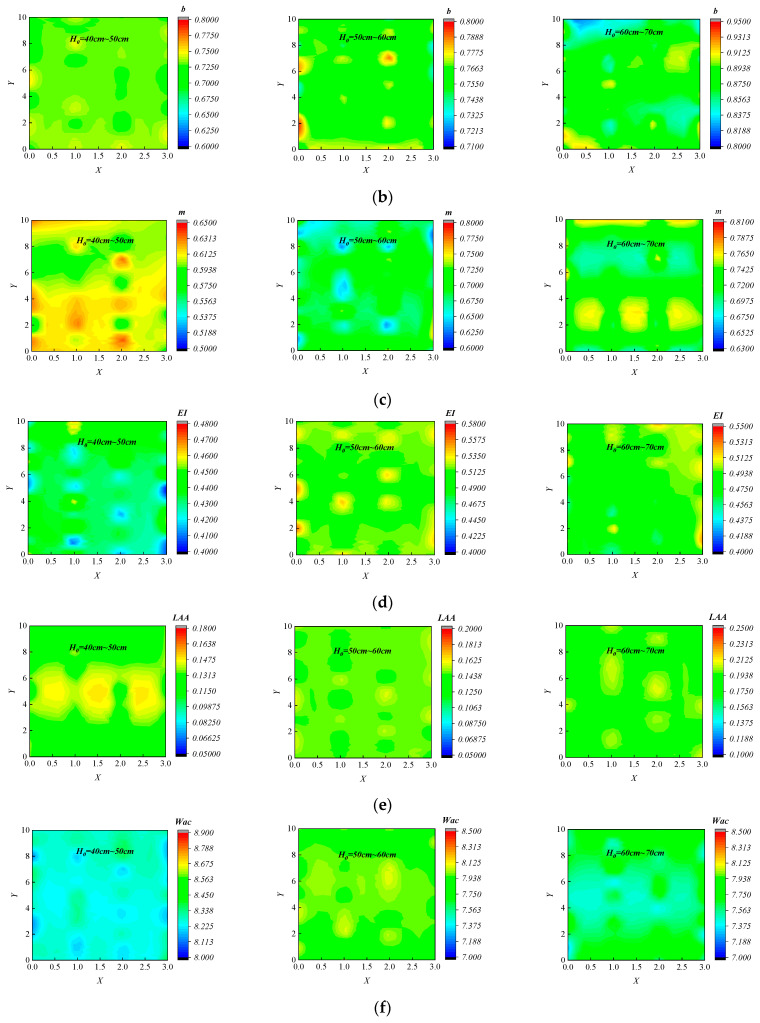

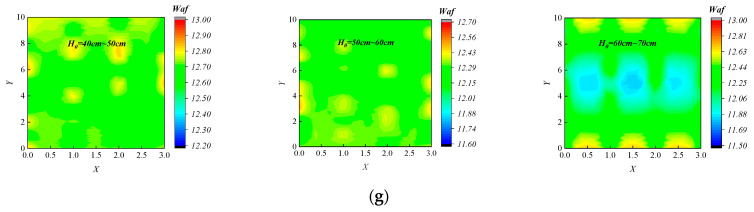
Full-section distribution results of filler parameters: (**a**) *d*_max_; (**b**) *b*; (**c**) *m*; (**d**) *EI*; (**e**) *LAA*; (**f**) *W*_ac_; and (**g**) *W*_af._

Taking the thickness of 40–50 cm as an example, the accuracy assessment results are shown in Table 4. The *MAE* and *MAPE* for each filler parameter are relatively small, with the maximum *MAE* being only 0.4073. This indicates that the Kriging interpolation algorithm used in this study has high accuracy [75,76], and it can effectively predict the spatial distribution of filler parameters. Furthermore, the interpolation results for filler parameters at three different thicknesses are shown in Figure 23. After obtaining the full-section parameters, they can be input into the compaction quality full-section assessment model to obtain the results of the full-section distribution interval for *ρ*_dmax_ and the results of the full-section compaction quality assessment.

### 4.3. Results of Full-Section Distribution Interval for ρ_dmax_

**Figure 24:**
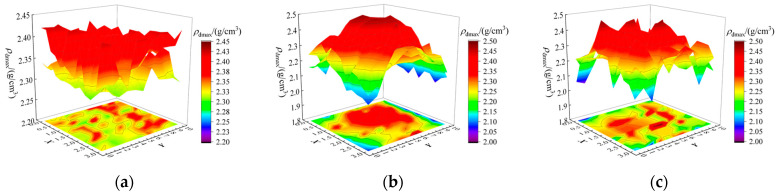
Results of the interval prediction for full-section *ρ_dmax_*: (**a**) 40~50 cm thickness; (**b**) 50~60 cm thickness; and (**c**) greater than 60 cm thickness.

The obtained spatial distribution of filler parameters across the full-section is input into the PSO-BPNN-AdaBoost model, yielding the full-section distribution of *ρ_dmax_* under different *H*_0_, as shown in Figure 24. It is obvious that under various *H*_0_, the upper limit of *ρ_dmax_* ranges between 2.30 and 2.45 g/cm^3^. In the test section with *H*_0_ = 40~50 cm, the uniform paving is easier to achieve due to the lower thickness, leading to a lower limit of *ρ_dmax_* ranging between 2.2 and 2.3 g/cm^3^. However, it is challenging to control the uniformity of the fillers with a larger thickness, which may cause a significant deviation of filler parameters from the design standards and result in a lower *ρ_dmax_* limit ranging between 2.0 and 2.3 g/cm^3^.

### 4.4. Results of Assessment for Full-Section Compaction Quality

**Figure 25:**
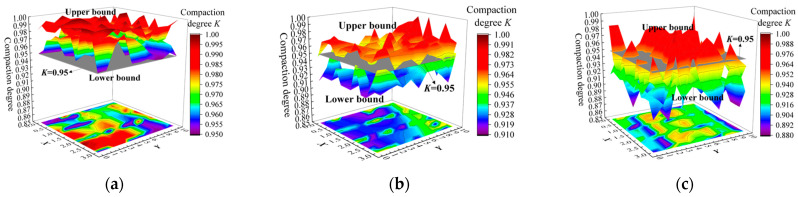
Results of the interval assessment for full-section compaction quality: (**a**) 40~50 cm thickness; (**b**) 50~60 cm thickness; and (**c**) greater than 60 cm thickness.

Moreover, by integrating the acquired full-section distribution interval results for *ρ_dmax_* into Equation (17), the full-section distribution interval results for *K* under different *H*_0_ are determined, as shown in Figure 25. The proposed method enables a visual, accurate, and comprehensive assessment of the compaction quality across the full-section of the subgrade. In the test section with *H*_0_ = 40~50 cm, the upper and lower bounds of the *K* both surpass 95% of the compaction quality standard, which indicates that choosing a paving thickness within the 40~50 cm range results in a well-compacted subgrade structure during field compaction.

However, when the paving thickness is set to 50~60 cm, the lower bound of the *K* fails to meet the 95% compaction quality standard. This indicates that in this thickness range, there may be areas that are not sufficiently compacted, and it cannot guarantee the compaction quality of the subgrade structure. Statistical analysis indicates that there exists 86.72% of the area at this thickness where the lower bound of the *K* value is less than 95%.

Similarly, when selecting a paving thickness above 60 cm, the upper bound of the *K* does not meet the 95% compaction quality standard, and there are 51.05% of areas below the 95% compaction quality standard. This indicates that in this thickness range, many areas are not sufficiently compacted, making it difficult to ensure the service performance of the subgrade. Hence, in the subsequent construction of this section, it is suggested to use a paving thickness of 40~50 cm to obtain a fully compacted subgrade, which can lay the foundation for ensuring the service performance of the subgrade.

## 5. Conclusions

This paper aims to address the mechanisms and methods for determining the *ρ_d_*_max_ still suffering from uncertainties, inefficiencies, and lack of intelligence. A novel method for full-section assessment of high-speed railway subgrade compaction quality based on ML-interval prediction theory is proposed. It is applied to determine the optimal paving thickness *H*_0_, within the station subgrade test section in the southwest region. The main conclusions are as follows:

1. The full-section assessment method for high-speed railway subgrade compaction quality, based on ML-interval prediction theory, not only quantifies the uncertainty in predicting *ρ_d_*_max_ using ML, but also provides results of assessment for full-section compaction quality, laying the foundation for ensuring the service performance of the subgrade.
2. The PSO-BPNN-AdaBoost model showed the highest prediction accuracy with an *R*^2^ of 0.9788, followed by the PSO-SVR-AdaBoost model (0.9453), and then the PSO-RF-AdaBoost model (0.9330). At the same time, the PSO-BPNN-AdaBoost model is chosen as the *POA* model for *ρ_d_*_max_ due to the PSO-BPNN-AdaBoost model also performing better in the error metrics *MSE*, *MAE*, and *MAPE*.
3. The proposed Bootstrap-POA-ANN interval prediction model for *ρ_d_*_max_ is capable of constructing clear and reliable prediction intervals and can effectively encompass the actual observed *ρ_d_*_max_ curve. Moreover, the optimal confidence level is determined to be 95% by combining the three metrics *PICP*, *MPIW*, and *CWC*.
4. The proposed compaction quality assessment model can provide the full-section distribution interval for *K*, and enable a visual, accurate, and comprehensive assessment of the compaction quality. The upper and lower limits of *K* for the 40~50 cm thickness exceed the 95% compaction quality standard, comparing the *H*_0_ of 50~60 cm and 60~70 cm. Hence, it is suggested to use the compaction thickness of 40~50 cm to ensure thorough compaction of the subgrade.

To further expand the conclusions of this paper, I will elaborate on two aspects: a discussion on broader implications and future research directions, and a discussion on potential integration into existing systems.

(1) Discussion on broader implications and future research directions

Based on the proposed assessment method for the subgrade compaction quality of high-speed railways, the compaction quality of the subgrade structure can be well ensured, laying the foundation for the service performance of the subgrade. The service performance of high-speed railway subgrades is easy due to the coupled effects of various external factors during operation, which may result in performance degradation that impacts operational safety. Subgrade settlement prediction serves as a crucial indicator for assessing the service performance of high-speed railway subgrades, offering insights into the advanced evolution trend of subgrade settlement. However, current research often remains at the prediction level or provides a ‘reference guide’ for assessing subgrade service performance without thoroughly exploring settlement prediction information. Furthermore, the subgrade settlement prediction process is influenced by various uncertainty factors. It is well known that assessments based on predictions with errors may introduce significant biases. As a result, enhancing the reliability of existing subgrade settlement predictions is essential.

**Figure 26:**
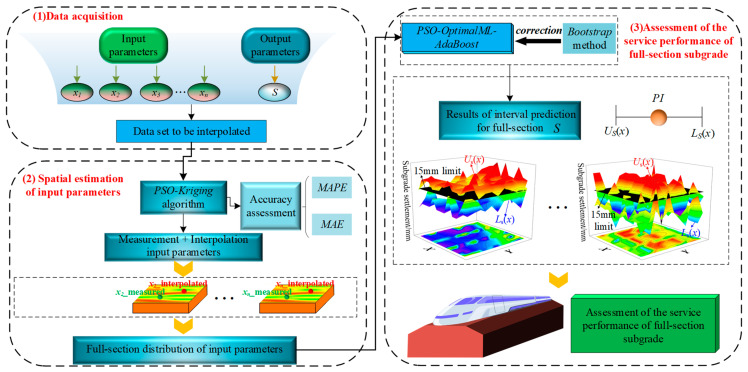
A framework for the full-section assessment of high-speed railway subgrade service performance based on ML-interval prediction theory.

In summary, a novel method for the full-section assessment of high-speed railway subgrade service performance is proposed based on the existing framework of this study and combined with the 15 mm settlement limit for high-speed railway subgrades. This method employs the ML-interval prediction theory, not only achieving the interval prediction of settlement across the subgrade full-section, but also enabling the comprehensive assessment of the subgrade service performance, as shown in Figure 26.

(2) Discussion on potential integration into existing systems

**Figure 27:**
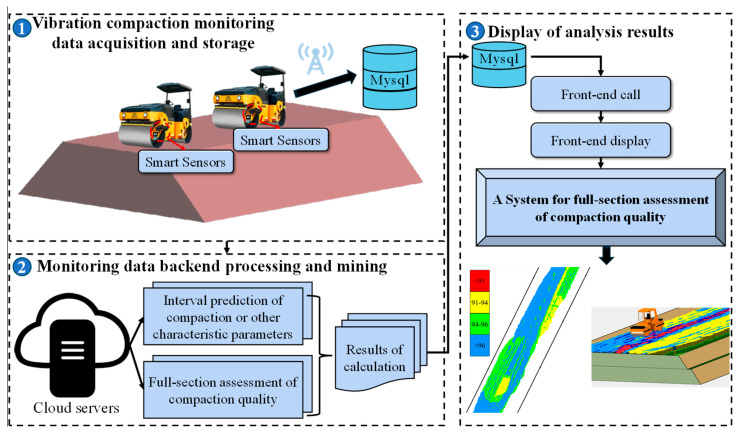
A full cross-section compaction quality assessment system.

Currently, based on the continuous testing indicator of compaction quality, continuous compaction control technology (ContinuousCompactionControl, CCC) has been developed by combining continuous testing technology with global positioning technology, computer technology, and communication technology. Through the CCC system and real-time monitoring, visualization display and data storage and analysis of the working status of the roller can be realized. In the future, the method proposed in this paper can be combined with an established continuous compaction control system to construct a full cross-section compaction quality assessment system. The logical structure of the system is shown in Figure 27, which includes three parts: vibration compaction monitoring data acquisition and storage, monitoring data backend processing and mining, and display of analysis results. Firstly, the data affecting the continuous compaction control indicator are obtained by various intelligent sensors deployed on the roller, transmitted to the system platform through the IOT system, and then stored in the Mysql database after processing. Secondly, the assessment results are calculated based on the computing model deployed in the cloud server and stored in the Mysql database. Finally, the front-end extracts the calculation results from the database for the presentation of the full-section compaction quality of the subgrade.

Another thing worth noting is the ethical implications of the research results obtained. The implementation of any new technology may pose certain risks and challenges. Hence, we need to thoroughly assess its possible ethical implications before promoting its application. I will elaborate on two aspects: a discussion on ethical implications, and a discussion of potential impacts on railway safety.

(1) Discussion on ethical implications

If the new method proposed in this paper is used reasonably, it can optimize the construction efficiency of the high-speed railway subgrade project to a certain extent by analyzing the compaction quality of the full-section in the compaction process and reducing the loss of compaction equipment. In addition, as the proposed methods fall into the data-driven category, they should be used with attention to privacy and data protection issues to ensure that the data are used legally and fairly.

(2) Discussion of potential impacts on railway safety

The high-speed railway subgrade compaction quality is controlled by *K*, with the *ρ*_dmax_ serving as a crucial indicator for its calculation. During the construction of high-speed railways, the method proposed in this paper can firstly determine a more accurate *ρ*_dmax_ so that the accuracy of *K* can be guaranteed. Secondly, the uncertainty in the process of obtaining the *ρ*_dmax_ is quantified by the *ML* algorithm and the interval prediction theory, and the degree of intelligence is improved, which makes the reliability and efficiency of the compaction obtained from the calculation guaranteed. Hence, the compaction quality and compaction efficiency can be improved to a certain extent by using the method proposed in this paper. The compaction quality affects the service performance of high-speed railroad subgrade, so the method in this paper has a certain guarantee for the subsequent safety of the railway.

## Figures and Tables

**Table 1:** Design of test parameters.

Grade	Moisture Content/%	Frequency/Hz	Mass of Eccentric Block/Kg	Eccentricity/cm
J1	3.6	40	2.4	1.91
J2	3.8	38	2.4	2.21
J3	4.0	34	2.4	2.66
J4	4.2	32	2.4	2.93
J5	5.4	26	2.4	4.64

**Table 2:** Results of accuracy assessment for point predictions.

Types of Metrics	Types of Algorithms		
	PSO-BPNN-AdaBoost	PSO-SVR-AdaBoost	PSO-RF-AdaBoost
R^2^	0.9788	0.9453	0.9330
EVS	0.9791	0.9468	0.9344
MSE (g·.cm^−3^)	0.0015	0.0039	0.0048
MAE (g·cm^−3^)	0.0167	0.0225	0.0295
MAPE (%)	0.9665	1.3552	1.7012

**Table 3:** Accuracy assessment results of different interpolation algorithms.

Types of Metrics	Types of Algorithms		
	Spline	IDW	Kriging
*MAE* (g·.cm^−3^)	0.0044	0.0037	0.0031
*MAPE* (%)	0.1916	0.1607	0.1357

**Table 4:** Accuracy assessment results of Kriging algorithm at 40~50 cm thickness.

Types of Metrics	Types of Filler Parameters						
	*d*_max_	*b*	*m*	*EI*	*LAA*	*W*_ac_	*W*_af_
*MAE* (g·.cm^−3^)	0.4073	0.0077	0.01498	0.0157	0.0061	0.0617	0.0681
*MAPE* (%)	1.0664	1.0548	2.5093	3.6163	4.8024	0.7455	0.5354

## Data Availability

The original contributions presented in the study are included in the article, further inquiries can be directed to the corresponding author/s.
